# 3-(Pyridin-4-ylmeth­oxy)phenol

**DOI:** 10.1107/S1600536810045800

**Published:** 2010-11-13

**Authors:** Liying Han, Hu Zang, Dajun Sun

**Affiliations:** a Department of Gynecology, The Second Hospital of Jilin University, Changchun 130041, People’s Republic of China; b Department of Orthopedics, The China–Japan Union Hospital of Jilin University, Changchun 130033, People’s Republic of China; c Department of Vascular Surgery, The China–Japan Union Hospital of Jilin University, Changchun 130033, People’s Republic of China

## Abstract

In the title compound, C_12_H_11_NO_2_, the phenolic ring is inclined at an angle of 32.70 (1)° with respect to the pyridine ring. In the crystal, inter­molecular O—H⋯N hydrogen bonds link the mol­ecules into *C*(11) chains along  [001].

## Related literature

For a related structure, see: Yumoto *et al.* (2008 ▶).
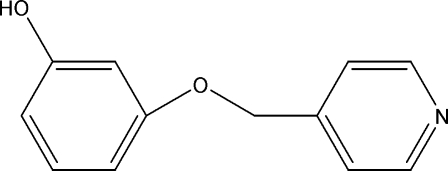


## Experimental

### 

#### Crystal data

C_12_H_11_NO_2_
*M* *_r_* = 201.22
Monoclinic, 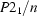

*a* = 6.6551 (6) Å
*b* = 9.1160 (8) Å
*c* = 17.0039 (15) Å
β = 100.501 (1)°
*V* = 1014.31 (16) Å^3^
*Z* = 4
Mo *K*α radiation
μ = 0.09 mm^−1^
*T* = 293 K
0.28 × 0.24 × 0.22 mm

#### Data collection

Bruker SMART APEXII CCD area-detector diffractometer
Absorption correction: multi-scan (*SADABS*; Sheldrick, 1996 ▶) *T*_min_ = 0.930, *T*_max_ = 0.980
5411 measured reflections
1981 independent reflections
1310 reflections with *I* > 2σ(*I*)
*R*_int_ = 0.098

#### Refinement

*R*[*F* ^2^ > 2σ(*F* ^2^)] = 0.041
*wR*(*F* ^2^) = 0.091
*S* = 0.89
1981 reflections
140 parameters
H atoms treated by a mixture of independent and constrained refinement
Δρ_max_ = 0.14 e Å^−3^
Δρ_min_ = −0.18 e Å^−3^

### 

Data collection: *APEX2* (Bruker, 2005 ▶); cell refinement: *SAINT* (Bruker, 2005 ▶); data reduction: *SAINT*; program(s) used to solve structure: *SHELXS97* (Sheldrick, 2008 ▶); program(s) used to refine structure: *SHELXL97* (Sheldrick, 2008 ▶); molecular graphics: *SHELXTL-Plus* (Sheldrick, 2008 ▶); software used to prepare material for publication: *SHELXL97*.

## Supplementary Material

Crystal structure: contains datablocks global, I. DOI: 10.1107/S1600536810045800/ng5062sup1.cif

Structure factors: contains datablocks I. DOI: 10.1107/S1600536810045800/ng5062Isup2.hkl

Additional supplementary materials:  crystallographic information; 3D view; checkCIF report

## Figures and Tables

**Table 1:** Hydrogen-bond geometry (Å, °)

*D*—H⋯*A*	*D*—H	H⋯*A*	*D*⋯*A*	*D*—H⋯*A*
O1—H1*A*⋯N1^i^	0.95 (2)	1.75 (2)	2.6991 (17)	174 (2)

Symmetry code: (i) 
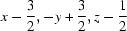
.

**Fig. 1.**
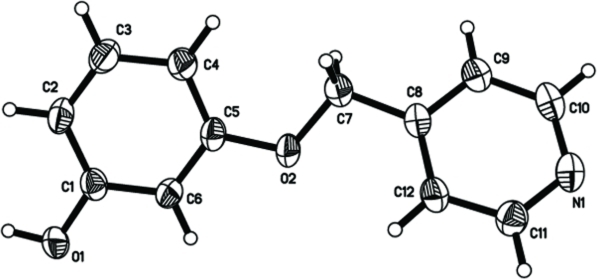
A view of the molecule of (I). Displacement ellipsoids are drawn at the 30% probability level.

## supplementary crystallographic information

### Comment

Pyridine and its derivatives represent one of the most active classes of compounds possessing a wide application of biological activities, such as stent in intestinal or biliary fields. During the past years, considerable efforts have been paid to demonstrate the efficacy of pyridine derivatives including antibacterial, antifungal, herbicidal, insecticidal and other biological activities. A new pyridine derivatives molecule is synthesized, with the aim of studying its single-crystal structure.

The title molecule (Fig. 1) consists of a phenol moiety (O1/C1—C6) and a methoxy moiety (O2/C7) attached to a pyridine ring (N1/C8—C12). The pyridine ring is inclined at an angle of 32.70 (1)° with the phenol ring. Bond lengths and angles are within normal ranges, and comparable to closely related structures (Yumoto *et al.*, 2008). In the crystal structure, the crystal packing is consolidated by intermolecular O1—H1A···N1 hydrogen bonds linking the molecules into one linear structure.

### Experimental

A mixture of 1,3-dihydroxybenzene (1.1 g, 10 mmol), 4-chloromethlpyridine hydrochloride (1.64 g, 10 mmol), and NaOH (1.6 g, 40 mmol) in acetonitrile (50 ml) was refluxed under nitrogen with stirring for 24 h. After cooling to room temperature, the reactant was filtered, and the residue was washed with acetonitrile several times. The mixed filtrate was slowly evaporated and colorless crystals were obtained.

### Refinement

All H-atoms bound to carbon were refined using a riding model with d(C—H) = 0.93 Å, *U*_iso_ = 1.2*U*_eq_ (C) for aromatic and 0.97 Å, *U*_iso_ = 1.2*U*_eq_ (C) for CH2 atoms. H atoms bonded to O atoms were located in a difference Fourier map.

### Crystal data

**Table:**

C_12_H_11_NO_2_	*F*(000) = 424
*M**_r_* = 201.22	*D*_x_ = 1.318 Mg m^−^^3^
Monoclinic, *P*2_1_/*n*	Mo *K*α radiation, λ = 0.71073 Å
Hall symbol: -P 2yn	Cell parameters from 1981 reflections
*a* = 6.6551 (6) Å	θ = 1.9–28.3°
*b* = 9.1160 (8) Å	µ = 0.09 mm^−^^1^
*c* = 17.0039 (15) Å	*T* = 293 K
β = 100.501 (1)°	Block, colorless
*V* = 1014.31 (16) Å^3^	0.28 × 0.24 × 0.22 mm
*Z* = 4	

### Data collection

**Table:**

Bruker APEX CCD area-detector diffractometer	1981 independent reflections
Radiation source: fine-focus sealed tube	1310 reflections with *I* > 2σ(*I*)
graphite	*R*_int_ = 0.098
φ and ω scans	θ_max_ = 26.0°, θ_min_ = 2.4°
Absorption correction: multi-scan (*SADABS*; Sheldrick, 1996)	*h* = −8→8
*T*_min_ = 0.930, *T*_max_ = 0.980	*k* = −9→11
5411 measured reflections	*l* = −15→20

### Refinement

**Table:**

Refinement on *F*^2^	Primary atom site location: structure-invariant direct methods
Least-squares matrix: full	Secondary atom site location: difference Fourier map
*R*[*F*^2^ > 2σ(*F*^2^)] = 0.041	Hydrogen site location: inferred from neighbouring sites
*wR*(*F*^2^) = 0.091	H atoms treated by a mixture of independent and constrained refinement
*S* = 0.89	*w* = 1/[σ^2^(*F*_o_^2^) + (0.0212*P*)^2^] where *P* = (*F*_o_^2^ + 2*F*_c_^2^)/3
1981 reflections	(Δ/σ)_max_ = 0.001
140 parameters	Δρ_max_ = 0.14 e Å^−^^3^
0 restraints	Δρ_min_ = −0.18 e Å^−^^3^

### Special details

**Table:**

Geometry. All e.s.d.'s (except the e.s.d. in the dihedral angle between two l.s. planes) are estimated using the full covariance matrix. The cell e.s.d.'s are taken into account individually in the estimation of e.s.d.'s in distances, angles and torsion angles; correlations between e.s.d.'s in cell parameters are only used when they are defined by crystal symmetry. An approximate (isotropic) treatment of cell e.s.d.'s is used for estimating e.s.d.'s involving l.s. planes.
Refinement. Refinement of *F*^2^ against ALL reflections. The weighted *R*-factor *wR* and goodness of fit *S* are based on *F*^2^, conventional *R*-factors *R* are based on *F*, with *F* set to zero for negative *F*^2^. The threshold expression of *F*^2^ > σ(*F*^2^) is used only for calculating *R*-factors(gt) *etc*. and is not relevant to the choice of reflections for refinement. *R*-factors based on *F*^2^ are statistically about twice as large as those based on *F*, and *R*- factors based on ALL data will be even larger.

### Fractional atomic coordinates and isotropic or equivalent isotropic displacement parameters (Å^2^)

**Table:**

	*x*	*y*	*z*	*U*_iso_*/*U*_eq_	
N1	0.7272 (2)	0.82817 (15)	1.01329 (9)	0.0343 (4)	
O1	−0.50233 (18)	0.67160 (13)	0.65242 (7)	0.0385 (3)	
O2	0.13905 (16)	0.82363 (12)	0.78584 (7)	0.0357 (3)	
H1A	−0.601 (3)	0.678 (2)	0.6047 (13)	0.073 (7)*	
C7	0.3149 (2)	0.91479 (18)	0.79342 (10)	0.0317 (4)	
H7A	0.2748	1.0170	0.7939	0.038*	
H7B	0.3829	0.8995	0.7482	0.038*	
C6	−0.1795 (2)	0.75328 (18)	0.71613 (9)	0.0296 (4)	
H6	−0.1743	0.6758	0.7520	0.036*	
C5	−0.0183 (2)	0.85103 (18)	0.72303 (9)	0.0295 (4)	
C12	0.3904 (2)	0.81807 (17)	0.93485 (10)	0.0320 (4)	
H12	0.2540	0.7921	0.9316	0.038*	
C8	0.4572 (2)	0.87800 (17)	0.86953 (10)	0.0271 (4)	
C11	0.5288 (3)	0.79727 (18)	1.00500 (11)	0.0345 (4)	
H11	0.4809	0.7596	1.0489	0.041*	
C1	−0.3484 (2)	0.77025 (19)	0.65603 (10)	0.0305 (4)	
C2	−0.3563 (3)	0.88767 (19)	0.60343 (10)	0.0356 (4)	
H2	−0.4703	0.9013	0.5635	0.043*	
C9	0.6626 (2)	0.90938 (18)	0.87728 (10)	0.0313 (4)	
H9	0.7143	0.9480	0.8344	0.038*	
C4	−0.0223 (2)	0.96653 (18)	0.67026 (10)	0.0348 (4)	
H4	0.0871	1.0313	0.6743	0.042*	
C3	−0.1947 (2)	0.98324 (19)	0.61087 (10)	0.0384 (5)	
H3	−0.2005	1.0613	0.5753	0.046*	
C10	0.7902 (2)	0.88286 (18)	0.94908 (10)	0.0346 (4)	
H10	0.9283	0.9043	0.9532	0.041*	

### Atomic displacement parameters (Å^2^)

**Table:**

	*U*^11^	*U*^22^	*U*^33^	*U*^12^	*U*^13^	*U*^23^
N1	0.0290 (8)	0.0365 (9)	0.0345 (9)	0.0046 (6)	−0.0018 (7)	−0.0058 (7)
O1	0.0276 (7)	0.0540 (8)	0.0305 (7)	−0.0094 (6)	−0.0038 (6)	0.0043 (6)
O2	0.0296 (7)	0.0405 (7)	0.0315 (7)	−0.0063 (5)	−0.0091 (5)	0.0046 (5)
C7	0.0266 (9)	0.0371 (10)	0.0304 (10)	−0.0015 (8)	0.0025 (8)	−0.0030 (8)
C6	0.0292 (9)	0.0352 (10)	0.0231 (9)	0.0006 (8)	0.0014 (8)	0.0026 (8)
C5	0.0266 (9)	0.0365 (10)	0.0231 (9)	0.0017 (8)	−0.0012 (7)	−0.0033 (8)
C12	0.0228 (9)	0.0375 (11)	0.0345 (10)	0.0018 (7)	0.0020 (8)	−0.0024 (8)
C8	0.0237 (9)	0.0280 (9)	0.0283 (9)	0.0033 (7)	0.0012 (7)	−0.0063 (7)
C11	0.0329 (10)	0.0388 (11)	0.0315 (10)	0.0025 (8)	0.0050 (8)	−0.0018 (8)
C1	0.0252 (9)	0.0401 (11)	0.0255 (9)	−0.0006 (8)	0.0029 (7)	−0.0043 (8)
C2	0.0312 (10)	0.0406 (11)	0.0307 (10)	0.0036 (8)	−0.0061 (8)	0.0026 (8)
C9	0.0287 (10)	0.0328 (10)	0.0323 (10)	0.0001 (7)	0.0055 (8)	−0.0063 (8)
C4	0.0325 (10)	0.0324 (10)	0.0364 (11)	−0.0041 (8)	−0.0016 (8)	0.0018 (8)
C3	0.0422 (11)	0.0319 (10)	0.0367 (11)	−0.0006 (8)	−0.0046 (9)	0.0057 (8)
C10	0.0243 (9)	0.0358 (10)	0.0419 (12)	0.0003 (8)	0.0015 (8)	−0.0112 (9)

### Geometric parameters (Å, °)

**Table:**

N1—C11	1.332 (2)	C12—C8	1.382 (2)
N1—C10	1.335 (2)	C12—H12	0.9300
O1—C1	1.3560 (19)	C8—C9	1.379 (2)
O1—H1A	0.95 (2)	C11—H11	0.9300
O2—C5	1.3756 (17)	C1—C2	1.390 (2)
O2—C7	1.4218 (18)	C2—C3	1.372 (2)
C7—C8	1.496 (2)	C2—H2	0.9300
C7—H7A	0.9700	C9—C10	1.375 (2)
C7—H7B	0.9700	C9—H9	0.9300
C6—C5	1.383 (2)	C4—C3	1.392 (2)
C6—C1	1.383 (2)	C4—H4	0.9300
C6—H6	0.9300	C3—H3	0.9300
C5—C4	1.381 (2)	C10—H10	0.9300
C12—C11	1.381 (2)		
			
C11—N1—C10	116.44 (15)	N1—C11—C12	123.70 (17)
C1—O1—H1A	113.5 (12)	N1—C11—H11	118.2
C5—O2—C7	117.50 (13)	C12—C11—H11	118.2
O2—C7—C8	109.17 (13)	O1—C1—C6	117.70 (16)
O2—C7—H7A	109.8	O1—C1—C2	122.82 (15)
C8—C7—H7A	109.8	C6—C1—C2	119.46 (16)
O2—C7—H7B	109.8	C3—C2—C1	119.52 (16)
C8—C7—H7B	109.8	C3—C2—H2	120.2
H7A—C7—H7B	108.3	C1—C2—H2	120.2
C5—C6—C1	120.27 (16)	C10—C9—C8	119.27 (16)
C5—C6—H6	119.9	C10—C9—H9	120.4
C1—C6—H6	119.9	C8—C9—H9	120.4
O2—C5—C4	124.39 (15)	C5—C4—C3	118.06 (16)
O2—C5—C6	114.70 (15)	C5—C4—H4	121.0
C4—C5—C6	120.91 (15)	C3—C4—H4	121.0
C11—C12—C8	119.11 (15)	C2—C3—C4	121.76 (17)
C11—C12—H12	120.4	C2—C3—H3	119.1
C8—C12—H12	120.4	C4—C3—H3	119.1
C9—C8—C12	117.64 (15)	N1—C10—C9	123.82 (16)
C9—C8—C7	119.78 (15)	N1—C10—H10	118.1
C12—C8—C7	122.54 (14)	C9—C10—H10	118.1

### Hydrogen-bond geometry (Å, °)

**Table:**

*D*—H···*A*	*D*—H	H···*A*	*D*···*A*	*D*—H···*A*
O1—H1A···N1^i^	0.95 (2)	1.75 (2)	2.6991 (17)	174 (2)

Symmetry codes: (i) *x*−3/2, −*y*+3/2, *z*−1/2.
